# Lower bumblebee colony reproductive success in agricultural compared with urban environments

**DOI:** 10.1098/rspb.2018.0807

**Published:** 2018-06-27

**Authors:** Ash E. Samuelson, Richard J. Gill, Mark J. F. Brown, Ellouise Leadbeater

**Affiliations:** 1 School of Biological Sciences, Royal Holloway University of London, Egham, UK; 2 Department of Life Sciences, Imperial College London, Silwood Park Campus, Ascot, UK

**Keywords:** urbanization, *Bombus terrestris*, reproductive success, land use, pollinator ecology, bee

## Abstract

Urbanization represents a rapidly growing driver of land-use change. While it is clear that urbanization impacts species abundance and diversity, direct effects of urban land use on animal reproductive success are rarely documented. Here, we show that urban land use is linked to long-term colony reproductive output in a key pollinator. We reared colonies from wild-caught bumblebee (*Bombus terrestris*) queens, placed them at sites characterized by varying degrees of urbanization from inner city to rural farmland and monitored the production of sexual offspring across the entire colony cycle*.* Our land-use cluster analysis identified three site categories, and this categorization was a strong predictor of colony performance. Crucially, colonies in the two clusters characterized by urban development produced more sexual offspring than those in the cluster dominated by agricultural land. These colonies also reached higher peak size, had more food stores, encountered fewer parasite invasions and survived for longer. Our results show a link between urbanization and bumblebee colony reproductive success, supporting the theory that urban areas provide a refuge for pollinator populations in an otherwise barren agricultural landscape.

## 1. Background

We are living in the ‘Urban Age’ [1]: over half the world's human population currently resides in cities [2] and an estimated threefold increase in global urban land cover is predicted between 2000 and 2030 [3]. Although urbanization has been shown to impact negatively upon species abundance and diversity for many taxa [4], some groups successfully exploit anthropogenic habitats [5] and there is evidence to place wild bees among this number. For example, areas subject to urban expansion have lost fewer pollinator species than agricultural areas over the past 80 years [6], and species richness has been found to be higher in urban than agricultural areas [7]. These community-level studies give reason to view urban environments as a potential refuge within barren agricultural landscapes, which have been associated with reduced floral resources [8] and exposure to environmental contaminants [9]. Yet, the crucial question of whether land use directly affects fitness—the ultimate driver of ecological success and evolutionary change—remains a largely neglected missing link in the correlations between urbanization and species abundance in both bees and other taxa [10–12].

Bumblebees comprise an important part of the pollinator community, but are currently subject to a multitude of threats that include changes in forage availability associated with land-use change [8] and pressure from emerging parasites and disease [13]. Alteration of floral resources is likely to be an important driver of urban effects on bees [14], with cities and towns often offering high floral abundance and diversity in the form of gardens and parks [15]. However, many horticultural plant varieties are unattractive to bees or invest energy in visual displays at the expense of reward provision [16], and competition may also affect forage availability: increased popularity of urban beekeeping has increased honeybee hive densities in urban areas [17], possibly increasing competitive interactions with wild bees [18]. Parasite prevalence has also been linked to urbanization, with higher parasite loads in urban areas reported in bumblebees [19,20]. Pesticide use has been identified as a threat to bees [9] and exposure may vary across degrees of urbanization [21]. In the context of this array of potentially interacting drivers of urban effects, it is not clear how inhabiting urban areas affects bumblebee success at the colony level. This is because ethical concerns preclude the release of reproductive offspring from commercially obtained bumblebee colonies [22–24], meaning that previous experiments have studied commercial colonies placed into the field only up to the very beginning of the period when reproductive offspring begin to emerge. Thus, while there is evidence that bumblebee colony early weight gain may be enhanced in suburban compared with agricultural areas based on studies of pre-reproductive colonies [25] (but cf. [24]), to date no study has monitored the critical, extensive reproductive period of the colony life cycle and thus assessed the effects of urbanization on lifetime reproductive success itself.

**Figure 1.**
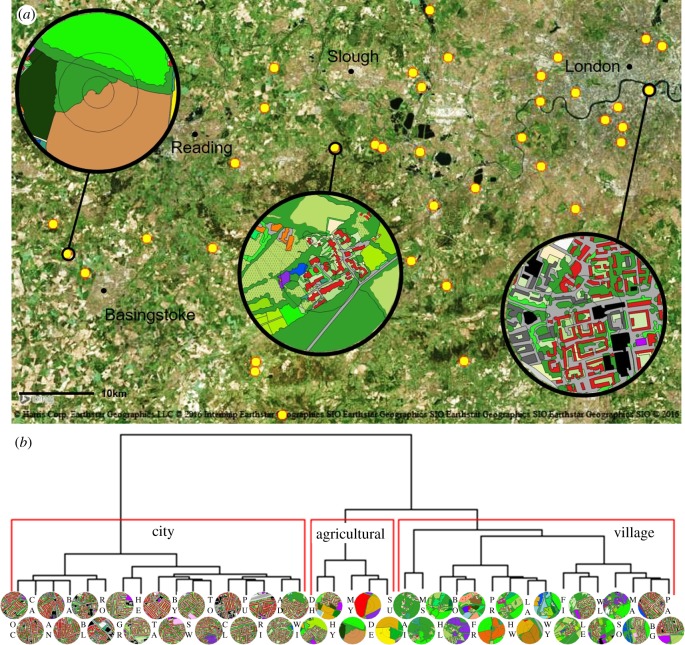
(*a*) Location of 38 sites in southeast England at which a *B. terrestris* colony was placed for up to 10 weeks from May to July. Inset circles show land-use classification at a 500 m radius for three typical sites (left to right: agricultural, village, city). (*b*) Cluster dendrograms of land use of 38 sites at a 500 m radius. Cluster analyses using Ward's method were performed on a set of principal components describing land use to group sites into categorical land-use types (red boxes). At the terminus of each branch the two-letter site name is given with an image of the GIS land classification (see electronic supplementary material, table S14 for colour key).

Here, we addressed this gap by rearing colonies from wild-caught queens to investigate the effect of urbanization on life history and reproductive output in the bumblebee *Bombus terrestris audax.* Using locally sourced queens allows ecologically relevant quantification of the impact of land use on locally adapted populations, in contrast to commercial bees that have been subjected to artificial selection [26] and may differ from locally adapted natural populations [24]. It also overcomes concerns associated with the use of commercial bees, including negative environmental impacts such as hybridization [22], pathogen spillover [23] and competition [24]. A crucial outcome is that colonies can be monitored for their entire reproductive lifetime. We selected 38 sites across central London, its suburbs and the surrounding agricultural land (figure 1*a*), and categorized each site based on land use through cluster analysis of principal components derived from 80 land-use variables. Through frequent censusing and sampling of colonies placed at these sites, we tracked for the first time the growth, reproductive output, nutritional status and parasite prevalence of each colony from eclosion of the first cohort of workers until the end of the colony life cycle. To our knowledge, this represents the first experimental study in any taxon to demonstrate a direct relationship between urbanization and reproductive success, with previous research typically employing an observational approach (e.g. [11,27]).

## 2. Material and methods

### (a) Bumblebee colonies

We collected 176 foraging *Bombus terrestris audax* queens in Windsor Great Park, Surrey, UK during March and April 2016. Queens were chilled and transported to the lab where they were immediately screened microscopically for the endoparasites *Nosema* spp., *Apicystis bombi*, *Sphaerularia bombi* and *Crithidia bombi,* by collecting faeces in a microcapillary (Baubrand Intramark, Wertheim, Germany) and examining the sample under ×400 magnification. Parasitized queens (*n* = 6) were excluded from the experiment. Queens were kept in clear acrylic rearing boxes until colony founding (see electronic supplementary material, methods for rearing protocol), after which they were rescreened and transferred to a wooden nest-box (W 280×L 320×D 160 mm) with a clear Perspex lid. Our final sample for placement in the field consisted of 43 colonies.

### (b) Field placement

We recruited 114 gardens and farms in southeast England (between central London and Basingstoke), of which 38 sites were selected across a region spanning inner city to countryside on the basis of distribution (greater than 1.5 km apart), land-use type representativeness and accessibility (figure 1*a*). This includes a range of urban and rural land-use types typical of western Europe [28], from central business district to suburban, villages and medium-intensity agriculture containing a mixture of grassland and arable fields. Predominant crop types in the agricultural areas were cereals and brassica crops. The wide range of urban land types contained within London means that it is representative of a range of different urban types displayed by smaller cities [29]. We placed colonies in the field in protective plastic field boxes during the first week of May 2016, randomized to land-use type according to initial colony size (see electronic supplementary material, methods). Colony placement was staggered over 6 days, with six or seven sites visited each day during daylight hours (8.00–20.00). Colony monitoring continued until moribundity (see below), which occurred for the last colony on 11 July.

### (c) Data collection

Site visits followed approximately the same order as the colony placement, with each site visited weekly during the hours of darkness (21.30–4.30) at the same time each week. We recorded the following data (see electronic supplementary material, methods for additional data): number of bees (average of three counts); queen status (alive, dead or absent); presence of nectar and pollen stores and presence and status of *Bombus vestalis* brood parasites (alive, injured or dead), which we removed to minimize *B. terrestris* queen death. To assess reproductive success, gynes were removed until one minute had passed with no gyne seen, and stored for later analysis. The same procedure was repeated for males, with sampling time capped at 15 min. Males are considered to leave the nest at 2–4 days old and gynes at 2–8 days old [30], so our weekly removal of males and gynes reflects natural conditions and is unlikely to have impacted the colony's production of future males and gynes. Weekly removal of reproductives allows calculation of total reproductive output over the colony life cycle rather than a snapshot as obtained from traditional colony dissection methods that are carried out at the earliest sign of reproductive emergence [25]. We removed one, three or five workers for later parasite analysis depending on colony size (less than 35, 35–50, greater than 50 workers, respectively), which were stored alive in vials for a maximum of 5 h before freezing at –20°C.

For the first two weeks, colonies in which the queen died (*n* = 5 of 43; one city, one village, three agricultural) were replaced with new colonies. Following this, colonies were removed from the field when moribund, defined as less than 10 workers remaining and queen death or less than three workers remaining with no queen death. Remaining workers were frozen at –20°C and dissected (see below). We obtained daily data for average temperature, average humidity and total rainfall for each site by downloading data from the weather station nearest to each site that had data for the full study period (www.wunderground.com).

### (d) Sample analysis

Up to three workers per colony per week were dissected. For each bee, the abdomen was placed in Ringer's solution and examined for the presence of conopid fly and braconid wasp larvae, and the larger tracheae for the tracheal mite *Locustacarus buchneri.* Sections of the Malpighian tubules, hindgut and fat body were removed, crushed and examined under ×400 magnification for the presence of the endoparasites *C. bombi, Nosema* spp. and *A. bombi.* Each slide was examined by two researchers. In addition, the ovary development of all collected workers (*n* = 393), and the body fat content of all workers, gynes (*n* = 46) and a random sample of max. 20 males per colony to limit workload (total *n* = 418) were assessed (see electronic supplementary material, methods).

### (e) Land-use classification

Following best practice in the field [31,32], we classified land use at multiple radii surrounding each site using GIS analysis, based on satellite imagery with additional ground-truthing for agricultural sites. Agricultural sites were surveyed because mass crop blooms may not be detected by satellite images taken outside the bloom period. The land-use classification protocol is described in full in [33] and is available as electronic supplementary material, methods. Briefly, buffers at radii of 750 m (*B. terrestris* typical foraging range [34–36]), 500 m, 250 m and 100 m (representing steps of spatial scales at which bees may interact with the surrounding land [12,33]) were generated around each site. Preliminary analysis showed that the majority of the response variables responded most strongly to land use at the 500 m radius, so this was selected as our primary land-use variable. Land-use patches were defined by drawing polygons in QGIS v. 2.16 and categorized visually to one of 80 land-use classes (electronic supplementary material, table S14) from satellite imagery and ground surveys carried out in May 2016.

We refined the classification to produce a single categorical land-use variable via an established three-step process [31]: (1) definition of land-use categories, (2) principal components analysis (PCA) on the categories and (3) cluster analysis based on the PCA output (electronic supplementary material, figure S2). Briefly, each land-use class was coded to one of eight categories (e.g. impervious surface, flower-rich habitat; electronic supplementary material, table S14) and the total area of each category within each site calculated. A PCA was then performed to reduce the dimensionality of the land-use variables, and cluster analysis (Ward's method) was performed on the first two principal components, which in combination captured approximately 85% of the variation (see below for loadings). Following [31,32,37], each cluster contained a minimum of five sites. Three clear clusters emerged (electronic supplementary material, figure S3a), comprising a group characterized by dense urban development (henceforth named ‘city’; *n* = 17), a group characterized by patches of housing surrounded by rural land (‘village’; *n* = 16) and a group dominated by agricultural fields (‘agricultural’; *n* = 5; figure 1*b*). Exploration of model fit confirmed that use of the clustered land-use categories to predict our main response variables explained more of the variance in our data than use of the PCs alone (electronic supplementary material, figure S3b), and comparison of models containing combinations of the PCs with those containing the clustered variable showed that, for all response variables, the clustered variable improved model fit (see electronic supplementary material, methods and table S11 for AIC values), justifying the necessity of the clustering step. Sites in the city cluster contained a mean 56.2% (±s.e.: 4.0%) impervious surface and 0.1 (±0.1)% agricultural land cover, while village and agricultural sites contained 13.8 (±3.7)% and 8.6 (±4.5)% impervious surface and 34.6 (±7.1)% and 71.2 (±11.5)% agricultural land cover, respectively.

### (f) Statistical analysis

For each analysis, we built a comparison set of models including the full model (for predictors; see below) and all subsets, including the basic model containing only the constant and residual variance (all-subset approach). We selected the model or set of models with the lowest AICc as the best-fitting model(s) [38]. Where several models were within two AICc units of the best model, model averaging was carried out to obtain parameter estimates derived from the best set of models including the basic model if applicable [39]. Final models were examined for spatial autocorrelation by using a Moran's I test on the residuals and graphically assessing the spatial pattern of residuals.

To analyse peak colony size, linear regression was carried out on log-transformed data. Total production of sexuals (gynes and males) was analysed using zero-altered negative binomial hurdle models, where the response is modelled as a binary process (production of sexuals) and a zero-truncated count process (total number of sexuals in colonies that produced sexuals) [40]. Binomial GAMs (allowing for a nonlinear effect of week) with site as a random effect were used to analyse the presence of nectar and pollen and ovary development. Queen survival, colony survival and onset of reproduction were subjected to survival analyses using non-parametric Cox proportional hazards models. Proportion of worker samples in each colony containing *Apicystis* and *Crithidia* were analysed using binomial GLMs. Male and worker fat content were analysed using Gaussian GAMs allowing for a nonlinear effect of week with site as a random effect. *Bombus vestalis* invasion as a binary response was modelled using binomial GLMs. One factor level (city) for this variable had perfect separation (only zeroes); to deal with this, three dummy observations were added for each land-use category with *B. vestalis* invasion set to one and weather variables set to whole-dataset means.

To investigate whether our results may have been driven by floral resource availability, we reanalysed the response variables that were found to be significantly affected by land use (reproductive output, peak colony size, colony survival, queen survival, presence of nectar stores and presence of pollen stores) using the proportion of flower-rich habitat as a predictor. We coded each land-use class as described above as flower-rich or flower-poor, based on reference to the literature (e.g. domestic gardens have been shown to support high floral diversity [15] and provide considerable resources to bees [41]) and on ground surveys in agricultural land to identify crop types and wild flower strips, and summed the area of flower-rich land-use patches to generate the proportion of flower-rich habitat at a 500 m radius for each site. Each response variable was analysed using this predictor as described in the paragraph above. All analyses were conducted in R v. 3.2.1 [42]; for packages see electronic supplementary material, methods.

## 3. Results

**Figure 2.**
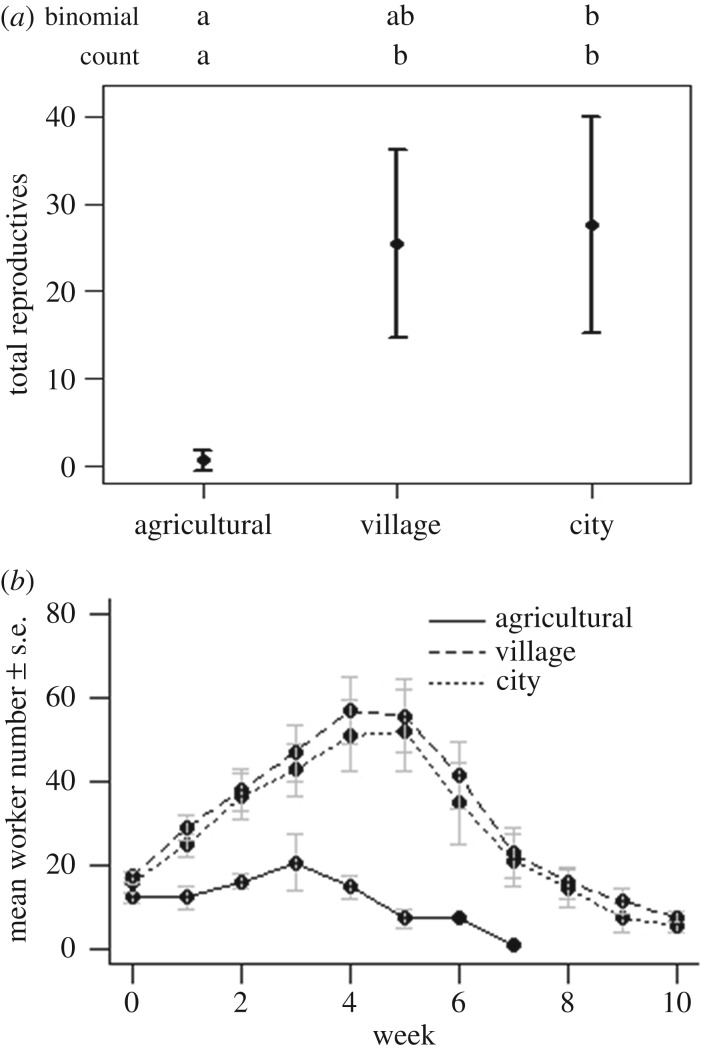
(*a*) Mean number of sexual offspring (gynes + males) with 95% confidence intervals (CIs) for colonies of *B. terrestris* in agricultural, village and city sites based on land use at a 500 m radius. Letters indicate significant differences between land-use types based on 95% CIs on parameter estimates from both the binomial (presence/absence of sexuals) and count (number of sexuals produced) components of a zero-inflated hurdle model. (*b*) Mean (±s.e.) colony size (number of bees) from weekly night-time bumblebee colony censuses. To analyse peak colony size, linear regression was carried out on log-transformed data.

**Figure 3.**
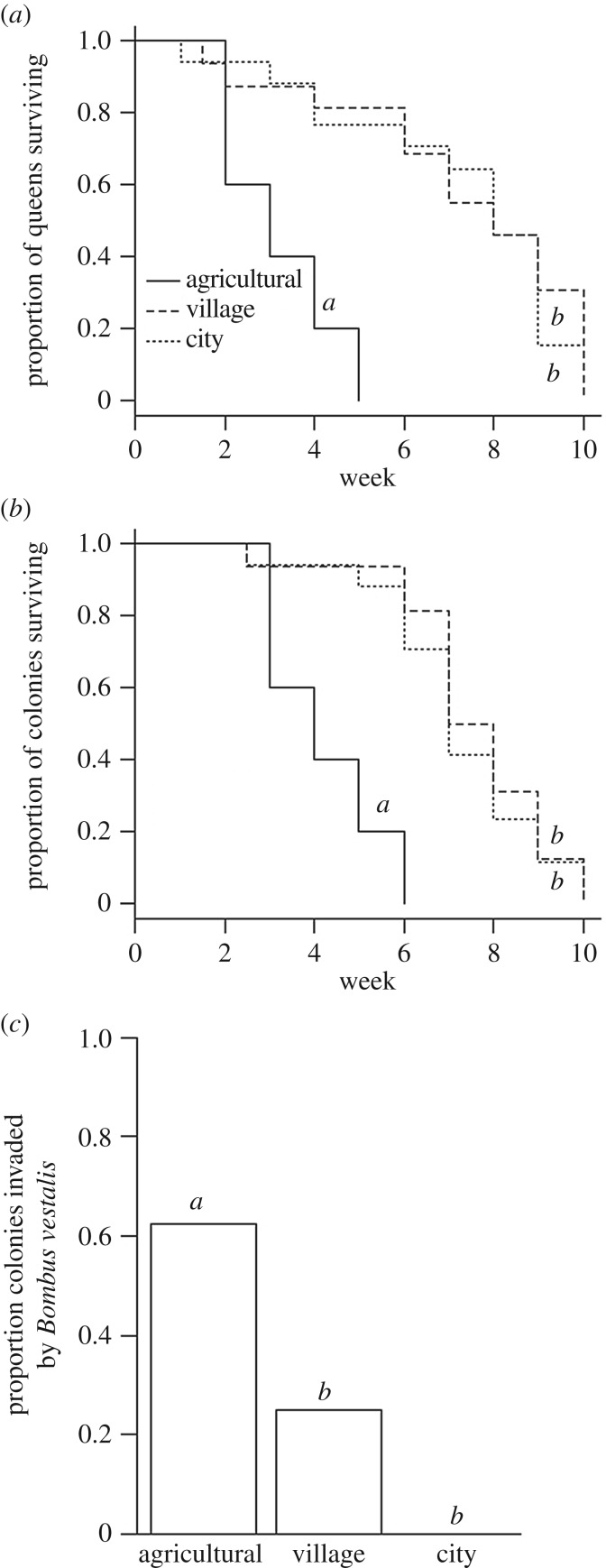
Kaplan–Meier curves of (*a*) queen survival and (*b*) colony survival for colonies of *B. terrestris* in agricultural, village and city sites based on land use at a 500 m radius. Each step in the Kaplan–Meier curves represents the week at which (*a*) queens died or (*b*) colonies were removed from the field; for example, all queens in agricultural sites had died by week 5. (*c*) Proportion of colonies invaded by *Bombus vestalis* in agricultural, village and city sites, analysed as a binary response. Letters indicate significant differences between land-use types based on 95% CIs on model-averaged parameter estimates from (*a*) and (*b*) Cox proportional hazards models and (*c*) binomial GLMs.

Land-use category strongly predicted the number of live sexual offspring (gynes and males) produced over the colony life cycle (figure 2*a*; electronic supplementary material, table S1a). Village colonies were significantly more likely to produce sexual offspring than agricultural colonies (model-averaged estimate (MAE): 2.853, 95% CIs: [0.327–5.378], electronic supplementary material, table S2a), and both city (MAE: 2.789 [0.799–4.778]), and village colonies (MAE: 2.566 [0.579–4.552]) produced significantly higher numbers of sexuals than their agricultural counterparts. Our data suggest that this effect may reflect both the build-up of a larger workforce and, relatedly, longer queen lifespans in village and city colonies. Both village and city colonies displayed significantly higher peak size (number of bees) than agricultural colonies (electronic supplementary material, table S1b; figure 2*b*; city MAE: 0.918 [0.194–1.641], village MAE: 1.047 [0.319–1.774]; electronic supplementary material, table S2b), and founding queens survived for longer (electronic supplementary material, tables S1c, S2c; figure 3*a*; MAE of hazard ratios (HR) relative to agricultural colonies: city: 0.149 [0.041–0.542]; village: 0.137 [0.039–0.488]). City and village colonies also took significantly longer to become moribund than agricultural colonies (city HR: 0.111 [0.031–0.396], village HR: 0.073 [0.019–0.271]; electronic supplementary material, table S1d; figure 3*b*). There was no significant effect of land-use on ovary development (see electronic supplementary material, results).

**Figure 4.**
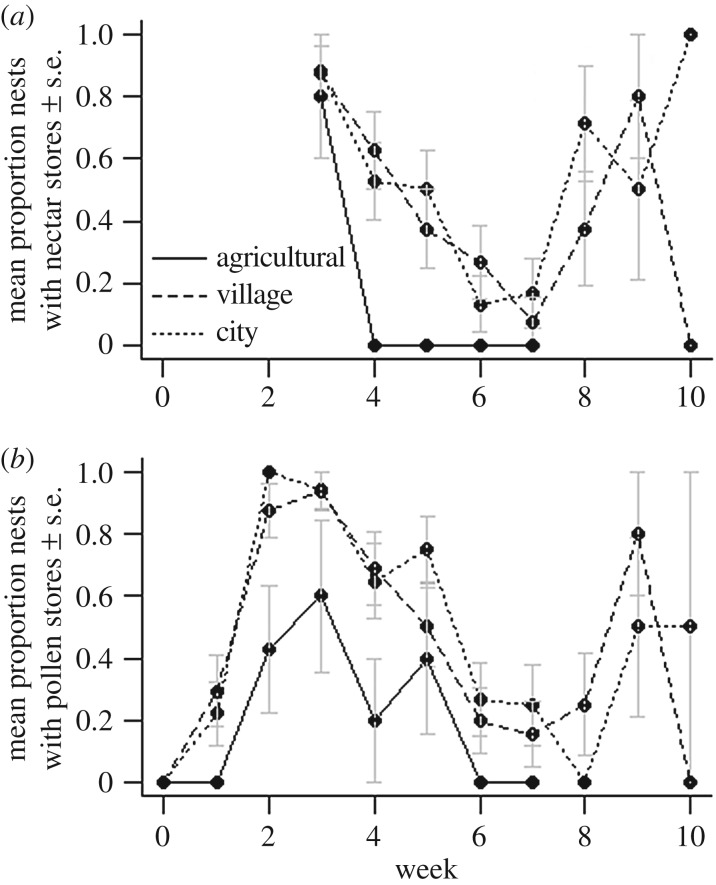
Mean (±s.e.) proportion of *B. terrestris* colonies containing (*a*) nectar and (*b*) pollen stores over 10 weeks in agricultural, village and city sites based on land use at a 500 m radius. Binomial GAMs allowing for a nonlinear effect of week with site as a random effect were used to analyse the presence of nectar and pollen. Nectar data were collected from week 3 due to provision of sucrose during week 1.

Agricultural colonies were found to contain less stored food than their city or village equivalents. Colonies in city (nectar MAE: 2.015 [0.520–3.509], electronic supplementary material, tables S1f, S2f; pollen MAE: 2.109 [1.045–3.173], electronic supplementary material, tables S1g, S2g) and village (nectar MAE: 1.902 [0.410–3.394]; pollen MAE: 2.038 [0.973–3.102]) land-use clusters were significantly more likely to contain nectar (figure 4*a*) and pollen (figure 4*b*) stores than agricultural colonies, in which we found almost no nectar stores and limited pollen after four weeks of development. We found no effect of land use on the fat content of workers or males (electronic supplementary material, tables S5b and S5c).

Land use had no effect on the prevalence of *Apicystis bombi* in colonies, although further analysis suggested that land use in the immediate area surrounding the colony may have an effect (see electronic supplementary material, results and table S5g). Similarly, there was no effect of land use on *C. bombi* presence (electronic supplementary material, table S5f). Only three bees were parasitized by *Syntretus* sp. (one city site and one village site), and no *N. bombi* or *Locustacaris buchneri* was found in any of our samples. The brood parasite *Bombus vestalis* was present in our study area, and hence we carefully monitored colonies to detect attempted parasite invasions. We recorded 14 invasion attempts by *B. vestalis* queens (max. 4 in a single colony). Land-use category was a significant predictor of the probability of an invasion attempt (electronic supplementary material, table S2 h), with city (MAE: −3.776 [−6.304 to −1.249]) and village (MAE: −2.943 [−5.444 to −0.442]) colonies being less likely to be invaded than agricultural colonies (electronic supplementary material, table S2h; figure 3*c*). Accordingly, we investigated the possibility that increased brood parasite invasions explain the poorer development of colonies in agricultural sites by performing a separate analysis in which three models were compared for each response variable: (1) the best model from the original analysis, (2) the same model but with parasite invasion events replacing land-use as a predictor, and (3) a model with both parasite invasion and land use. For all variables, the model containing land use only or land use and invasions fitted the data better than the model containing invasions alone (i.e. had a lower AICc value; electronic supplementary material, table S9). In other words, although parasite invasions explain some of the variance in our data, land use influences colony performance irrespective of invasion status.

Our land classification protocol [43] allows investigation into the aspects of the land use that may underlie the effects found, by examining the variables that contribute to the clustering of land-use types. High domestic infrastructure, impervious surface and road cover, and low agricultural land cover contributed strongly to principal component (PC) 1 (eigenvalue score greater than 0.4 or less than −0.4 [44]), while PC2 was defined by high tree cover and low open and flower-rich habitat cover (electronic supplementary material, table S10). The city cluster was characterized by positive scores on PC1 (mean 2.00 ± s.e. 0.07) and near-zero scores on PC2 (−0.27 ± 0.17), suggesting a highly urbanized semi-open land type; the village cluster had medium negative scores on PC1 (−1.40 ± 0.37) and positive PC2 scores (0.94 ± 0.37), suggesting low-intensity urbanization with moderate tree cover; the agricultural cluster had low PC1 scores (−2.33 ± 0.64) and low PC2 scores (−2.08 ± 0.32), suggesting open land with very little urbanization and high agricultural cover (electronic supplementary material, figure S3a). Analysis of the PCs suggested it was the combination of both attributes of the land use that drove the effects seen (see electronic supplementary material, methods and table S12 & S13 for results of these analyses). Investigation of the effect of the proportion of flower-rich habitat on the response variables as a possible key driver of the results showed no significant effect (electronic supplementary material, methods; tables S3 and S4).

## 4. Discussion

Our results demonstrate a direct association between urbanization and higher reproductive output in a key insect pollinator, *B. terrestris*. We found increases in reproductive output, colony growth and food stores as well as lower brood parasitism by *B. vestalis* in colonies placed in urbanized areas compared to sites dominated by agriculture. Previous research has described correlative evidence for higher abundance of bees (e.g. [45]) and higher bumblebee nest densities [41,46] in urban areas, but whether this may be driven by migration between land-use types or effects of land use on population dynamics has remained unclear [47]. Our experimental design, whereby colonies reared from wild-caught queens were placed in different land-use types over the full colony life cycle in order to measure reproductive output, provides evidence for a causal link between reproductive success and urbanization, elucidating a potential mechanism behind these observed differences in pollinator populations between urban and rural areas. Our use of colonies established from locally sourced queens gives our findings direct ecological relevance to the impacts of land-use change on wild bumblebee populations.

We employed a high-resolution approach to measuring reproduction, collecting almost all males and gynes present in the nest at weekly night-time inspections, over the entire colony life cycle from first worker emergence to moribundity. This builds on traditional methods of dissecting nests at the very onset of reproduction [25,48,49], capturing a higher proportion of the total reproductive output and allowing worker and male production to be distinguished [10], which may explain our detection of a strong effect of urban land use on reproduction in contrast to previous studies [25,50,51]. Furthermore, consideration of asymmetrical reproductive investment in gynes and males means our results are potentially conservative. Gyne production requires greater resource investment than male production [52], and in our study, agricultural colonies failed to produce even a single gyne. Gyne production is likely to have a particularly strong effect on population dynamics, as queens hibernate and found new colonies [53], so our findings suggest that agricultural populations may not be self-sustaining [54]. Queens of common bumblebee species may migrate long distances [55], raising the possibility that cities may act as a source of new queens to replenish such agricultural population sinks and therefore support the pollination of crops in agriculturally intense landscapes.

Parasite pressure presents a significant emerging threat to wild bee populations [13] and previous research has provided evidence for a link between land-use and parasite prevalence in bumblebees [19,20]. However, no effect of land use was found on *C. bombi* presence, and levels of *N. bombi, Syntretus* sp. and *L. buchneri* were either zero or too low for analysis. Conversely, invasions by the brood parasite *B. vestalis* were strongly affected by land use, with higher invasion rates in agricultural and village colonies than city. This may reflect lower *B. vestalis* abundance or even complete absence in the urban areas studied, although surveys have recorded the species in cities (e.g. [56]). Alternatively, stronger colonies in city sites may have been more able to resist invasion [57], or volatiles from colonies may have been masked by air pollution [58], rendering them more difficult to locate [59]. Reductions in forage availability in modern agricultural landscapes have been identified as a potential major driver of bee population declines [8]. Accordingly, we found less stored pollen and nectar in agricultural colonies than in city or village colonies, suggesting forage availability may be a contributing factor to poor performance at agricultural sites. This is consistent with evidence from honeybees, where urbanization has been shown to have a positive effect on food storage [60] (but cf. [61]), and supports research suggesting modern agricultural land provides insufficient forage resources for bees [8].

Investigation into the underlying attributes of our land-use classification indicates that it appears to be the shared attributes of high agricultural cover and low urbanization that group the poorly performing colonies in our study. A reasonable hypothesis from previous research showing higher colony weight gain in suburban areas than agricultural [25] would be that low-intensity urban areas are most valuable to bee populations due to the combination of abundant gardens and proximity to semi-natural habitat; our finding that colonies in densely urbanized areas performed similarly to those in lower-intensity urbanization nonetheless fails to support this. We found no direct effect of the proportion of flower-rich habitat surrounding colonies on colony success. However, this may reflect the fact that fine-resolution floral abundance surveys, taking into account floral density and species identity, are not possible in urban areas due to access restrictions to gardens. Future research could aim to investigate forage provision in urban areas using modelling approaches [62] to further assess floral availability as a driver in urban habitats. Floral factors differing between agricultural and built-up areas that may have contributed to a reduced ability to collect food may include the spatial distribution and composition of flower-rich patches [16,63], the duration for which they are available [63] or potential effects of environmental contaminants on foraging behaviour [64].

Exposure to agrochemicals has been shown to have an impact on colony function and success in bumblebees [49,64], including reproductive output [49] and parasite prevalence [65], and high levels of pesticide contamination are often found in both crop and wild flower resources in agricultural areas [66]. There is evidence that bees in urban areas may be subject to lower pesticide exposure [21] (but cf. [67]), offering another possible mechanism for our findings of lower colony success in agricultural areas. Ground surveys of the agricultural sites in this experiment showed a variety of crops in the surrounding farmland, with one site near a field of oilseed rape. This may represent a route of pesticide exposure [68], although the study took place after the EU moratorium restricting neonicotinoid use in flowering crops [69]. However, neonicotinoids may remain in the soil and the nectar and pollen of non-target plants for prolonged periods following use on nearby crops [70], and other pesticides may also negatively affect bees [64]. In general, fields around the agricultural sites were more commonly arable than pasture, compared to the village sites which more often contained pasture and woodland in undeveloped areas, providing the potential for different pesticide exposure between these land-use types, and the high incidence of gardens and parks in city areas may expose bees to a different suite of horticultural pesticide applications, about which little is known [70]. Our findings highlight that the question of how bee exposure to pesticides varies with urbanization is a major knowledge gap that requires exploration.

We show for the first time that the reproductive output of *B. terrestris* colonies placed in built-up areas is higher than in agricultural areas, suggesting that the current urban expansion may have positive consequences for generalist bumblebee species. Our findings suggest that abundance and diversity differences found in previous studies [71] may be driven by a direct impact of land use on fitness, rather than migration between land-use types, and support the growing evidence that some types of agricultural land represent a barren landscape for pollinators [8,12]. Given that agricultural land is the most common primary land use in Europe [72], our finding that urban areas are linked to higher reproductive success suggests that developed land may provide a refuge for bumblebee populations within a landscape dominated by intensive farming.

## Supplementary Material

Supplementary Material

## Supplementary Material

Supplementary Tables

## Data accessibility

Raw data are archived in Dryad (http://dx.doi.org/10.5061/dryad.c68cj62) [73].
