# Supplementary material for: Lower bumblebee colony reproductive success in agricultural compared with urban environments
Source: Proc Biol Sci. 2018 Jun 27;285(1881):20180807. doi: 10.1098/rspb.2018.0807 (PMC6030522; doi:10.1098/rspb.2018.0807)
Supplement: Supplementary Material [file rspb20180807supp1.docx]

**Lower bumblebee colony reproductive success in agricultural compared to urban environments**

**Supplementary Material**

Ash E. Samuelson^a*^, Richard J. Gill^b^, Mark J. F. Brown^a^ & Ellouise Leadbeater^a^

^a^ School of Biological Sciences, Royal Holloway University of London, Egham, United Kingdom

^b^ Department of Life Sciences, Imperial College London, Silwood Park campus, Ascot, United Kingdom

*Corresponding author: ash.samuelson.2014@live.rhul.ac.uk

**Appendix S1: Supplementary Methods**

***Bumblebee colony rearing***

Queens were kept in clear acrylic rearing boxes (W 67 x L 127 x D 50; Allied Plastics, Kingston, UK) with a plastic perforated mesh base (The Plastic Shop, Coventry, UK). Each queen was provided with a gravity feeder (Savic, Kortrijk, Belgium) containing 25% inverted sucrose solution (Thorne, Windsor, UK) and a pollen ball formed of finely ground pollen (Biobest, Westerlo, Belgium) and 25% sucrose solution. These were changed weekly unless the queen was incubating the pollen ball or eggs were present, in which case an additional pollen ball was added. Gravity feeders were refilled every two days. Bees were kept in the dark or under red light at 26°C and 50-60% RH.

We monitored colony founding, excluding queens that had not laid eggs after 8 weeks from the experiment (n=51). Colonies that hatched 10 workers were transferred into a wooden nest box (W 280 x L 320 x D 160mm) with a clear Perspex lid. During transfer, queens again underwent faecal screening for parasites and colonies with parasitized queens excluded from the experiment (n=2). Colonies were kept in the wooden nest boxes in the lab for 7-10 days before field placement; mean time (± SE) between queen collection and placement in the field was 35.45 ± 0.58 days.

***Field placement***

We advertised the study on social media, requesting gardens and farms in South-East England (between central London and Basingstoke) with side access and not accessed regularly by children under 16 or dogs. For the purposes of colony randomisation, sites were initially classified to four crude land-use types based on visual classification using Google Earth: urban (U), suburban (S), village (V) or agricultural (A); these classifications were not used for further analysis. Colonies containing between 10 and 25 workers were ordered by worker number and assigned first to land-use type in cyclical order (USVASVAUVAUSAUSV...)., and then to individual sites in visit order, with colonies containing more workers assigned to earlier visited sites to maximise equality of colony size at time of placement.

We installed colonies in the presence of the site owner, who was given an information sheet and asked to sign a consent form before colony placement. Wooden nest boxes were placed inside protective field boxes, consisting of a plastic storage box (W 440 x L 710 x D 310mm; Really Useful Box, Kingston, UK) lined with insulation wrap (Thermawrap, Creswell, UK) inside the walls and lid and aluminium foil on the base (Fig. S1), and connected to the outside via a 10cm black plastic pipe (diameter: 32mm) inserted through a 40mm hole in the field box. To protect against badger damage, field boxes were fixed to the ground using screw or groundhog pegs and secured with a ratchet strap. We placed field boxes under a tree or bush for shade, away from paths or benches and in a location receiving morning sun and midday shade as indicated by the site owner. No sucrose or pollen was fed to the colonies throughout the experiment, except that each colony initially received a small gravity feeder containing 150ml 25% inverted sucrose solution to reduce any risk of mortality following transfer, which was removed after one week.

***Data collection and analysis***

The field boxes contained EM-30KAM scales connected to AD-1688 weighing data loggers (A&D Instruments, Abingdon, UK), which stood on a ceramic floor tile to provide a hard surface. Nest boxes were placed on the scales to allow continuous weight recording. On arrival at each visit, we replaced the scale head unit containing the display and battery with a charged unit, weighed a known 1kg weight and then recorded the weight of the nest box before carrying out any manipulations. The weight data are not presented here, as humidity from the colonies caused the measurements to be unreliable.

In addition to the data reported in the main text, we also collected the following data: photograph of the nest before and after removing the wax cover, number of gyne larvae and pupae, wax cover (full, partial or absent), presence of open or closed egg towers as an indicator of competition point (1) and presence of dead *B. terrestris* queens, indicating usurpation attempts. A pollen sample was taken from one pollen pot if present.

During dissection of collected workers, ovaries were removed and the presence of developed oocytes was recorded. If present, the length of the terminal oocyte of each of the 8 ovarioles was measured to assess ovary development (2, 3). Ovary development was modelled as a binary response (presence/absence of at least one developed oocyte) using binomial GAMs allowing for a non-linear effect of week with site as a random effect. The effect of queen presence on ovary development was analysed with binomial GLMMs with site as a random effect (week was not included as it was highly collinear with queen presence).

The body fat content of all collected workers (n=393) and gynes (n=46), and a random sample of max. 20 males per colony to limit workload (total n=418), was assessed (4). For each bee, we measured thorax width (between tegulas (wing joints)) as the mean of three digital calliper measurements (accuracy = 0.01mm) and wet mass using a precision balance (accuracy = 0.0001g) before drying for 72h at 70°C. The dry mass of the whole bee and abdomen alone were measured before immersing the abdomen in 4ml diethyl ether for 24h to extract the fat. The abdomen was rinsed with fresh ether, dried for 72h and weighed again. The difference between the weights before and after ether extraction represents the amount of fat; relative fat content is expressed as the ratio of fat (mg) to thorax width (mm). Only two males were collected from agricultural colonies (compared to 179 in village and 189 in city) so these were excluded from analysis of the effect of land-use at a 500m radius on male size and fat content.

***Land-use classification***

The full land-use classification protocol is as follows. Sites were located on Google Earth version 7.1.5.1557 and imported into QGIS version 2.16. Buffers were generated around each site at four radii (see below). Polygons were drawn around each visually-identified land-use patch at 1:5000m (agricultural areas) or 1:2500m (built-up areas) zoom at a resolution separating individual buildings, fields and gardens. Each polygon was assigned to one of 34 initial land-use classes (e.g. house, residential garden, arable field, hedgerow; for full list see Appendix S3) using Bing Aerial satellite imagery. Ground surveys were carried out in May 2016 at all sites which contained agricultural land within a 750m radius (n=19) to further classify fields by crop grown, bloom stage and presence of wildflower strips and other floral resources. This resulted in a total of 80 land-use classes.

To refine the classification to produce the categorical land-use variables used in the analysis, we carried out a three-step process following Owen et al. (2006) (5): 1) definition of land-use categories, 2) PCA on the categories and 3) cluster analysis to generate a categorical land-use variable (Fig. S2). To do this, the total area of each land-use class was calculated around each site. Eight land-use categories were defined: impervious surface (including built area), flower-rich habitat, domestic infrastructure, garden, tree cover, agricultural land, open land and road (excluding vegetated verges). Each of the 80 land-use classes was coded according to whether it belonged to each category based on *a priori* knowledge and reference to the literature (e.g. “house” was coded as belonging to impervious surface and domestic infrastructure, and “garden” to flower-rich habitat (6, 7) , domestic infrastructure and open land; Table S14), allowing calculation of the proportion of each category at each radius. For example, the total area of woodland, hedgerow and freestanding trees was summed and divided by the total area of the circle to generate the proportion of tree cover. A PCA was then performed to reduce the dimensionality of the land-use variables. Two principle components captured approximately 85% of the variation, “PC1” and “PC2” (Table S10).

A cluster analysis using Ward’s method was carried out on the principle components to group sites with similar land-use attributes, to allow for non-linearity in the effect of degree of urbanisation and to incorporate land-use information into a single variable capturing both principle components. Similar land classification methods typically select optimum numbers of clusters using an ad hoc minimum group size based on practicality and geographical relevance (5, 8, 9); following this approach we split clusters so that each cluster contained a minimum of five sites.

We investigated whether the clustered land-use variable generated by our land classification protocol or the principle components (PCs) themselves better fit our data by performing an additional analysis on our main response variables (reproductive output, colony size, colony survival, queen survival, nectar stores and pollen stores) in which we compared a model containing the clustered land-use variable with models containing all possible combinations of the PCs including their interaction. We found that for all response variables, the model with the clustered land-use variable had the lowest AIC (Table S11), justifying the use of the clustered variable in the main analysis.

To investigate whether one or both of the PCs were driving the effects of land-use on the response variables, we performed a full analysis on each of the main response variables (see *Statistical Analysis* section in main text for regressions used) using a model selection approach to compare models containing combinations of the PCs and the weather covariates and performing model averaging on the optimal model set (Tables S12 & S13). No clear pattern emerged, with little evidence that one PC is more important than the other, with the combination of both often key. It is the combination of the PCs which drives the clustering, as suggested by the grouping of the PC scores (Fig S3a), so these findings further support use of the clustered land-use variable.

*Radius selection*

Land-use was initially classified for four radii around each site: 750m, 500m, 250m and 100m; hereafter R750, R500, R250 and R100. This allows for the analysis to detect the nearest spatial scale at which colonies respond, which may vary depending on the response (10). A PCA was carried out for all radii; for R750, R500 and R250, two principle components captured 85% of the variation and three for R100. Cluster analysis on the principle components produced one categorical land-use variable for each site ranging from the landscape to the local scale: R750: Urban, Rural; R500: City, Village, Agricultural (Fig. 1b in main text); R250: Built-up, Open; R100: Dense housing, Sparse housing, Wooded, Fields.

We carried out an initial analysis of each dependent variable by fitting a full model for each radius containing the relevant land-use variable (R750, R500, R250 or R100), weather covariates (temperature, humidity and rainfall) and week (for time series) and compared the models using Akaike Information Criteria corrected for small sample sizes (AICc). Models containing R500 fit the data best for the majority of the variables (Table S8) suggesting that colonies responded most strongly to landscape at a 500m radius. We therefore selected this as our main land-use variable for the full analysis.

***Statistical analysis***

Dependent variables were either analysed as multiple observations per colony over time (e.g. male size) with site as a random effect, or at the colony level (e.g. total number of sexual offspring). Daily weather records were averaged (temperature and humidity) or summed (rainfall) over each week of the experiment. At five sites colonies became moribund within the first two weeks and were replaced; to ensure data for both the original and replacement colonies were included in the analysis data for these sites were pooled and an average taken. Final models were validated graphically to assess fit and check that assumptions had been met (11). For the statistical analysis the R packages survival (12), MuMIn (13), plotrix (14), lattice (15), mgcv (16), VGAM (17), nlme (18), lme4 (19), effects (20), pscl (21), ade4 (22), spdep (23), ncf (24) were used for model fitting and producing graphs.

**Appendix S2: Supplementary Results & Discussion**

Classification of land-use around the colonies showed that sites clustered into three distinct groups: agricultural, village and city. Land surrounding agricultural sites was dominated by fields, while village sites were characterised by housing in the immediate vicinity of the colony within a rural landscape and city sites consisted of dense inner-city urban land. Colonies in the two land-use types containing built-up areas- village and city- grouped together, performing better overall than agricultural colonies. This suggests a positive impact of local urban patches on colony success, with wider landscape context contributing less to the effect of land-use.

Land-use at the 500m radius did not affect the size of males produced (Table S5d), but further analysis including land-use at other radii showed that local habitat at a 100m radius did affect male size (Table S7a, Fig. S5a), with significantly larger males in colonies in wooded land than fields (MAE: 0.279 [0.050 – 0.508], Table S7a) and a trend towards larger males in sparse housing (MAE: 0.192 [-0.052 – 0.436]) and dense housing (MAE: 0.185 [-0.037 – 0.406]), an effect potentially mediated by resource availability (25, 26). However, we found no effect of land-use on size of workers (Table S5e). Onset of reproduction was not significantly affected by land-use (Table S5a).We dissected samples of workers to assess ovary development, and found the optimal model set for ovary development contained models including land-use (Table S1e), with a non-significant trend (i.e. 95% CIs cross zero) towards a higher proportion of bees in agricultural colonies displaying developed ovaries than city (MAE: -1.739 [-3.830 – 0.351]) or village (MAE: -1.912 [-4.002 – 0.178], Fig. S4; Table S2e). There was a strong effect of queen presence on ovary development (ΔAICc to basic model = 12.71), with bees in colonies where the queen was alive being less likely to have developed oocytes (MAE: -1.241 [-1.877 – -0.604]).

In general, parasite levels were low compared to other studies (27-30), possibly due to an unusually wet spring, which may have impeded transmission of pathogens on flowers (31, 32). Land-use at the 500m radius did not affect the presence of the parasite *Apicystis bombi* in colonies (Table S5g). Further analysis showed that local habitat at the 100m radius did have an effect (Table S6b), but did not show a consistent response to increasing urbanisation, with a lower proportion of colonies containing the parasite in dense housing (MAE: -2.756 [-4.882 – -0.630]) or wooded (MAE: -2.147 [-3.790 – -0.505]) sites than in sparse housing sites (Table S7b, Fig. S5b). Little is known about *A. bombi* transmission (33), but the effect of land-use on its prevalence may be mediated by bumblebee densities (27) or spillover from commercial colonies (34).

Total invasion rates by *Bombus vestalis* (21% of colonies) were lower than reported rates in previous field studies which ranged from 30%-100% (35-39), although several field studies report no brood parasite invasions (40, 41) suggesting high natural variation in this response. Although invasions by *B. vestalis* explain some of the variance in our data, our analysis suggests land-use influences colony performance irrespective of invasion status. Invasions were not included in the main analysis to generate parameter estimates as this was highly collinear with land-use.

Rainfall positively affected male size (MAE: 0.006 [0.002 – 0.010], Table S2b), peak colony size (MAE: 0.013 [0.000 – 0.026], Table S2c.) and marginally increased colony survival (Hazard ratio: 0.977 [0.956 – 0.999], Table S2e). Rainfall also marginally negatively affected presence of pollen in the nest (MAE: -0.026 [-0.053 – 0.000]).

Lower reproductive output and colony growth in agricultural colonies may have been partially driven by the fact that agricultural colonies lost their queen earlier on average, halting gyne and worker production and restricting male production to workers (42). Correspondingly, there was a trend towards bees in agricultural colonies being more likely to have developed ovaries, which was strongly linked to queen absence. Queen loss may have been driven in turn by nutritional stress or parasitism (30, 42, 43).


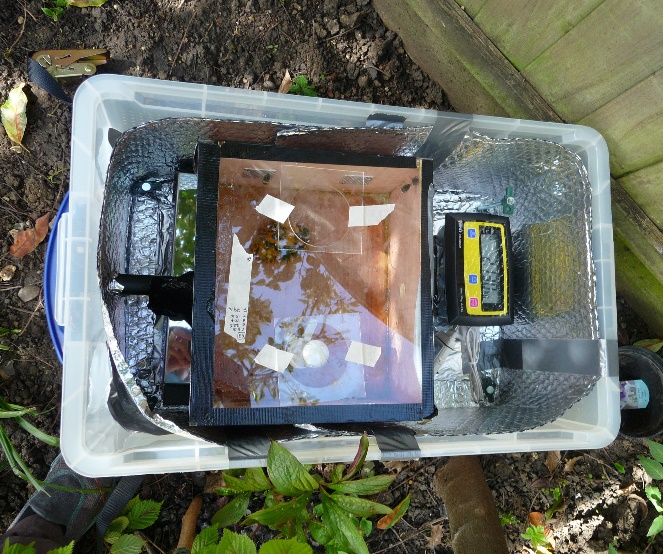

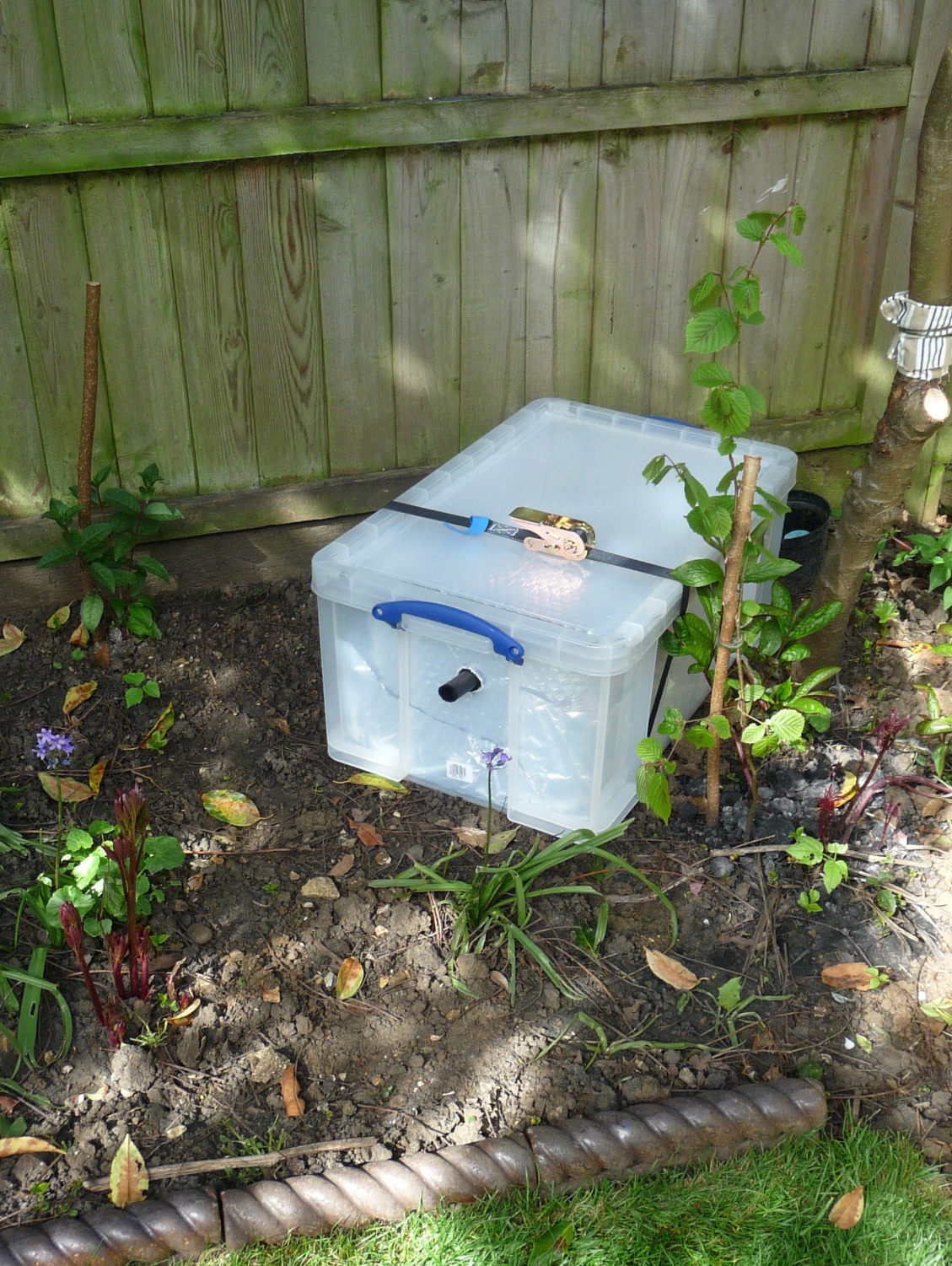


**Figure S1**. Insulated field box containing a young *Bombus terrestris* colony at time of placement in the field. Boxes contained a data logger connected to scales on which the colony box was placed; the colony was connected to the outside by a plastic pipe.

**
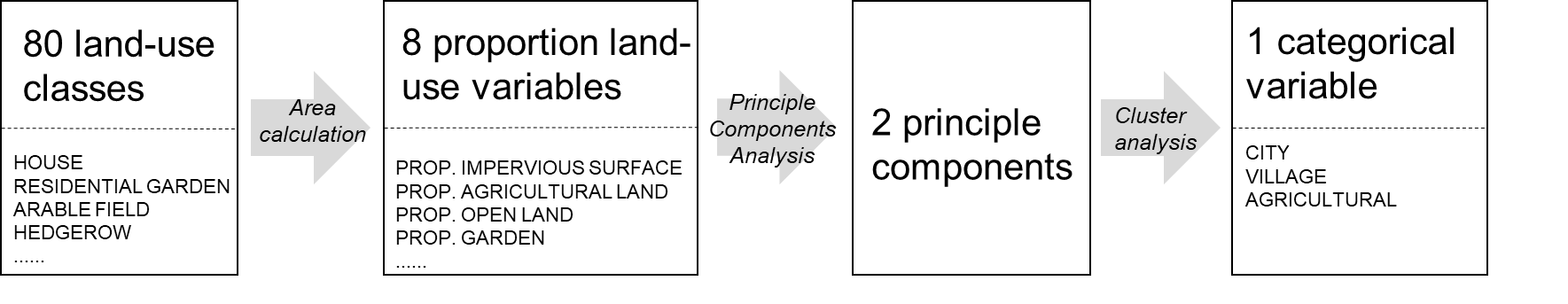
**

**Figure S2:** Overview of the methods used to classify land-use at 38 sites across a gradient of urbanisation in SE England.

**
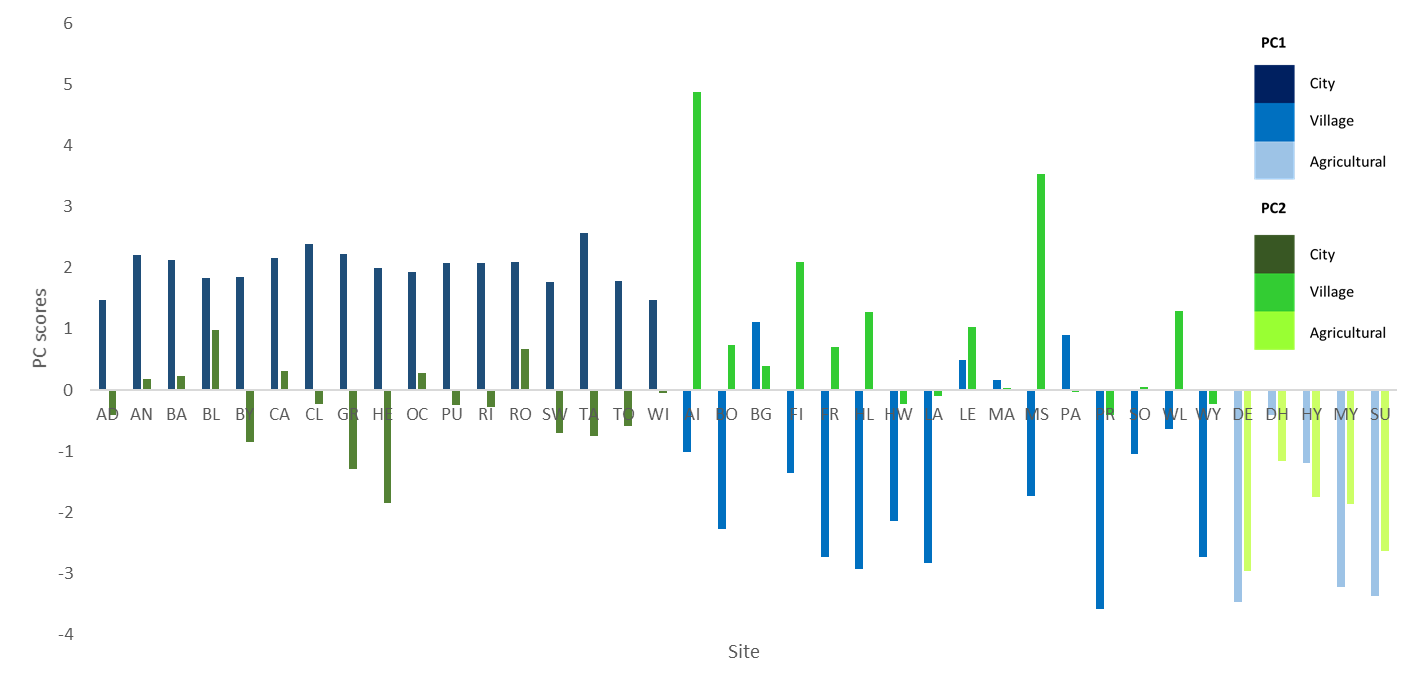
**

b

a


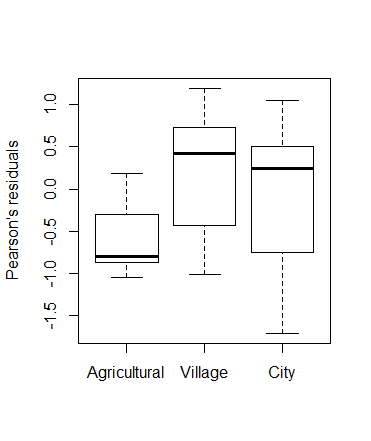


**Figure S3. a)** Scores on two principal components, PC1 and PC2, that captured approximately 85% of the variation in a principle component analysis (PCA) performed on land-use variables classified in a 500m radius around each study site (two-letter codes). The clustering of the three land-use types generated from subsequent cluster analysis (“City”, “Village” and “Agricultural”), is illustrated in the grouping of PC scores (City: dark blue/green; Village: bright blue/green; Agricultural: pale blue/green). The “City” cluster is typified by a positive score on PC1 and neutral score on PC2, “Village” by positive to neutral PC1 and negative PC2, and “Agricultural” by negative PC1 and PC2. b) Plot of Pearson’s residuals for model validation purposes of a model including PC1 and PC2 as predictors of colony size by land-use cluster from the original land classification (not included in this model). This shows variation following the clusters that is not explained by the model using the PCs alone, justifying the further step of cluster analysis.


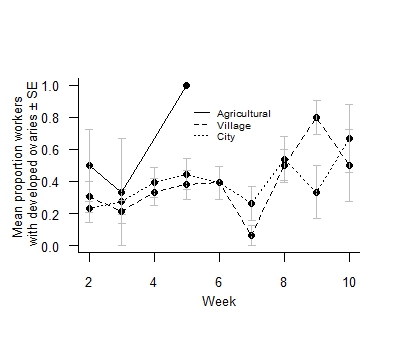


**Figure S4.** Mean (± SE) proportion of workers with developed ovaries by week from samples of workers collected for dissection.

**
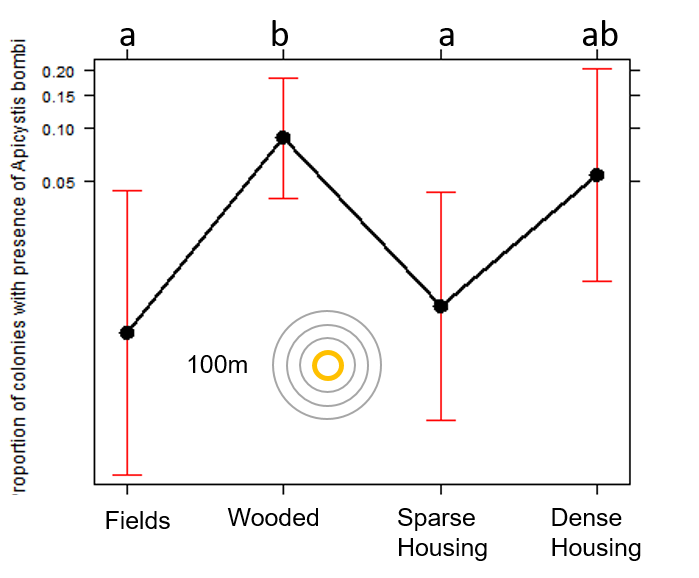
**

**Figure S5** a). Mean thorax width of males with 95% CIs for colonies of *B.terrestris* in fields, wooded, sparse housing and dense housing sites based on land-use at a 100m radius. Letters indicate significant differences between land-use types based on 95% CIs on model-averaged parameter estimates. b) Effects plot from a binomial GLM showing the effect of land-use at a 100m radius on presence of the parasite *Apicystis bombi* in colonies of *B. terrestris* placed in sites across an urbanisation gradient. Error bars indicate 95% confidence intervals and letters indicate significant differences between land-use types based on 95% confidence intervals on parameter estimates.

**References**

1. Duchateau MJ & Velthuis HHW (1988) Development and reproductive strategies in *Bombus terrestris* colonies. *Behaviour* 107(3/4):186-207.

2. Evans LJ, Raine NE, & Leadbeater E (2016) Reproductive environment affects learning performance in bumble bees. *Behavioral Ecology and Sociobiology* 70(12):2053-2060.

3. Amsalem E & Hefetz A (2010) The appeasement effect of sterility signaling in dominance contests among *Bombus terrestris* workers. *Behavioral Ecology and Sociobiology* 64(10):1685-1694.

4. Brown MJF, Loosli R, & Schmid‐Hempel P (2000) Condition‐dependent expression of virulence in a trypanosome infecting bumblebees. *Oikos* 91(3):421-427.

5. Owen S*, et al.* (2006) Urban land classification and its uncertainties using principal component and cluster analyses: A case study for the UK West Midlands. *Landscape and Urban Planning* 78(4):311-321.

6. Loram A, Thompson K, Warren PH, & Gaston KJ (2008) Urban domestic gardens (XII): the richness and composition of the flora in five UK cities. *Journal of Vegetation Science* 19(3):321-330.

7. Goulson D*, et al.* (2010) Effects of land use at a landscape scale on bumblebee nest density and survival. *Journal of Applied Ecology* 47(6):1207-1215.

8. Bunce RGH, Barr CJ, Clarke RT, Howard DC, & Lane AMJ (1996) Land Classification for Strategic Ecological Survey. *Journal of Environmental Management* 47(1):37-60.

9. Hall O & Arnberg W (2002) A method for landscape regionalization based on fuzzy membership signatures. *Landscape and Urban Planning* 59(4):227-240.

10. Moreira EF, Boscolo D, & Viana BF (2015) Spatial heterogeneity regulates plant-pollinator networks across multiple landscape scales. *PloS ONE* 10(4):e0123628.

11. Zuur AF, Hilbe J, & Ieno EN (2013) *A Beginner's Guide to GLM and GLMM with R: A Frequentist and Bayesian Perspective for Ecologists* (Highland Statistics, Newburgh).

12. Therneau T (2015) _A Package for Survival Analysis in S_), 2.38.

13. Barton K (2015) MuMIn: Multi-Model Inference), 1.14.0.

14. Lemon J (2006) Plotrix: a package in the red light district of R. *R-News* 6(4):8-12.

15. Sarkar D (2008) *Lattice: Multivariate Data Visualization with R.* (Springer, New York).

16. Wood SN (2011) Fast stable restricted maximum likelihood and marginal likelihood estimation of semiparametric generalized linear models. *Journal of the Royal Statistical Society: Series B (Statistical Methodology)* 73(1):3-36.

17. Yee TW (2010) The VGAM package for categorical data analysis. *Journal of Statistical Software* 32(10):1-34.

18. Pinheiro J, Bates D, DebRoy S, Sarkar D, & R Development Core Team (2015) _nlme: Linear and Nonlinear Mixed Effects Models_.), R package version 3.1-121.

19. Bates D, Mächler M, Bolker B, & Walker S (2014) Fitting linear mixed-effects models using lme4. *arXiv preprint arXiv:1406.5823*.

20. Fox J (2003) Effect displays in R for generalised linear models. *Journal of statistical software* 8(15):1-27.

21. Zeileis A, Kleiber C, & Jackman S (2008) Regression models for count data in R. *Journal of statistical software* 27(8):1-25.

22. Dray S & Dufour A-B (2007) The ade4 package: implementing the duality diagram for ecologists. *Journal of statistical software* 22(4):1-20.

23. Bivand R & Piras G (2015) Comparing implementations of estimation methods for spatial econometrics. (American Statistical Association).

24. Bjornstad ON (2016) ncf: Spatial Nonparametric Covariance Functions.), R package version 1.1-7.

25. Ribeiro M (1994) Growth in bumble bee larvae: relation between development time, mass, and amount of pollen ingested. *Canadian Journal of Zoology* 72(11):1978-1985.

26. Sutcliffe G & Plowright R (1988) The effects of food supply on adult size in the bumble bee *Bombus terricola* Kirby (Hymenoptera: Apidae). *The Canadian Entomologist* 120(12):1051-1058.

27. Goulson D, Whitehorn P, & Fowley M (2012) Influence of urbanisation on the prevalence of protozoan parasites of bumblebees. *Ecological Entomology* 37(1):83-89.

28. Whitehorn PR, Tinsley MC, Brown MJ, Darvill B, & Goulson D (2011) Genetic diversity, parasite prevalence and immunity in wild bumblebees. *Proceedings of the Royal Society of London B: Biological Sciences* 278(1709):1195-1202.

29. Imhoof B & Schmid-Hempel P (1999) Colony success of the bumble bee, *Bombus terrestris*, in relation to infections by two protozoan parasites, *Crithidia bombi* and *Nosema bombi*. *Insectes Sociaux* 46(3):233-238.

30. Rutrecht ST & Brown MJF (2008) The life-history impact and implications of multiple parasites for bumble bee queens. *International Journal for Parasitology* 38(7):799-808.

31. Durrer S & Schmid-Hempel P (1994) Shared use of flowers leads to horizontal pathogen transmission. *Proceedings of the Royal Society of London B: Biological Sciences* 258(1353):299-302.

32. Graystock P, Goulson D, & Hughes WO (2015) Parasites in bloom: flowers aid dispersal and transmission of pollinator parasites within and between bee species. *Proc. R. Soc. B*, (The Royal Society), p 20151371.

33. Meeus I, Brown MJ, De Graaf DC, & Smagghe G (2011) Effects of invasive parasites on bumble bee declines. *Conservation Biology* 25(4):662-671.

34. Graystock P*, et al.* (2013) The Trojan hives: pollinator pathogens, imported and distributed in bumblebee colonies. *Journal of Applied Ecology* 50(5):1207-1215.

35. Sladen FWL (1912) *The Humble-Bee* (Cambridge University Press, Cambridge).

36. Carvell C, Rothery P, Pywell RF, & Heard MS (2008) Effects of resource availability and social parasite invasion on field colonies of *Bombus terrestris*. *Ecological Entomology* 33(3):321-327.

37. Pelletier L & McNeil JN (2003) The effect of food supplementation on reproductive success in bumblebee field colonies. *Oikos* 103(3):688-694.

38. Müller CB & Schmid-Hempel P (1992) Correlates of reproductive success among field colonies of *Bombus lucorum*: the importance of growth and parasites. *Ecological Entomology* 17(4):343-353.

39. Erler S & Lattorff HMG (2010) The degree of parasitism of the bumblebee (*Bombus terrestris*) by cuckoo bumblebees (*Bombus (Psithyrus) vestalis*). *Insectes sociaux* 57(4):371-377.

40. Goulson D, Hughes W, Derwent L, & Stout J (2002) Colony growth of the bumblebee, *Bombus terrestris*, in improved and conventional agricultural and suburban habitats. *Oecologia* 130(2):267-273.

41. Williams NM, Regetz J, & Kremen C (2012) Landscape‐scale resources promote colony growth but not reproductive performance of bumble bees. *Ecology* 93(5):1049-1058.

42. Goulson D (2003) *Bumblebees: their behaviour and ecology* (Oxford University Press, Oxford).

43. Frehn E & Schwammberger K-H (2001) Social parasitism of *Psithyrus vestalis* in free-foraging colonies of *Bombus terrestris* (Hymenoptera: Apidae). *Entomologia Generalis* 25(2):103-105.
